# Flowmotion Monitored by Flow Mediated Skin Fluorescence (FMSF): A Tool for Characterization of Microcirculatory Status

**DOI:** 10.3389/fphys.2020.00702

**Published:** 2020-06-19

**Authors:** Joanna Katarzynska, Tomasz Cholewinski, Leslaw Sieron, Andrzej Marcinek, Jerzy Gebicki

**Affiliations:** ^1^Angionica Ltd., Lodz, Poland; ^2^Institute of Applied Radiation Chemistry, Lodz University of Technology, Lodz, Poland

**Keywords:** microcirculation, skin flowmotion, response to hypoxia, FMSF technique, NADH fluorescence

## Abstract

Oscillations in the microcirculation, known as flowmotion, are a well-recognized characteristic of cutaneous blood flow. Since flowmotion reflects the microcirculatory status of the vascular system, which is very often impaired in many diseases and disorders, a quantitative assessment of skin flowmotion could potentially be used to screen for early symptoms of such conditions. In this study, skin flowmotion was monitored using the Flow Mediated Skin Fluorescence (FMSF) technique. The flowmotion parameter was used for quantitative assessment of basal flowmotion both at rest (FM) and during reperfusion [FM(R)] following the post-occlusive reactive hyperemia (PORH). The study population was composed of healthy volunteers between the ages of 30 and 72 (*n* = 75). The FM parameter showed an inverse dependence relative to age, while the FM(R) parameter was inversely correlated to blood pressure. The FM(R) parameter reflects the strong effect of hypoxia on flowmotion, which is mainly due to increased myogenic activity in the vessels. The FMSF technique appears to be uniquely suited for the analysis of basal flowmotion and the hypoxia response, and may be used for the characterization of microcirculatory status.

## Introduction

Oscillations in the microcirculation, known as flowmotion, are a well-recognized characteristic of cutaneous blood flow (Nilsson and Aalkjaer, 2003; Rossi et al., 2006b). The mechanistic aspects of flowmotion have been the object of extensive study (Aalkjær et al., 2011; Cole et al., 2019). The use of Laser Doppler Flowmetry (LDF) allows for the semi-quantitative characterization of changes in human cutaneous blood flow (Stefanovska et al., 1999; Kvandal et al., 2006). Such analysis in the frequency domain reveals that blood flow oscillations fit into several periodic activities, classified as follows: endothelial NO-independent (<0.0095 Hz); endothelial NO-dependent (0.0095–0.021 Hz); neurogenic (0.021–0.052 Hz); myogenic (0.052–0.15 Hz); respiratory (0.15–0.62 Hz), and cardiac (0.62–2.00 Hz) (Bernjak et al., 2008; Ticcinelli et al., 2015; Clough et al., 2017).

There is substantial evidence that impaired flowmotion can be a symptom of various diseases and disorders, including diabetes, cancer, and cardiovascular diseases, as well as neurodegenerative and autoimmune diseases (Schmidt-Lucke et al., 2002; Bari et al., 2005; Rossi et al., 2013; Bruning et al., 2015; Tikhonova et al., 2016; Mizeva et al., 2017; Pedanekar et al., 2019; Sorelli et al., 2019). However, quantitative analysis of changes in flowmotion caused by different pathologies is not always reliable, due to the substantial noise associated with LDF measurements.

Our new technique, known as Flow Mediated Skin Fluorescence (FMSF), is based on monitoring the intensity of NADH fluorescence emitted from skin tissue on the forearm (Piotrowski et al., 2016; Katarzynska et al., 2019b). Since NADH fluorescence is sensitive to the supply of oxygen to the epidermis via skin microcirculation, it can be used to indirectly monitor skin blood flow.

**FIGURE 1:**
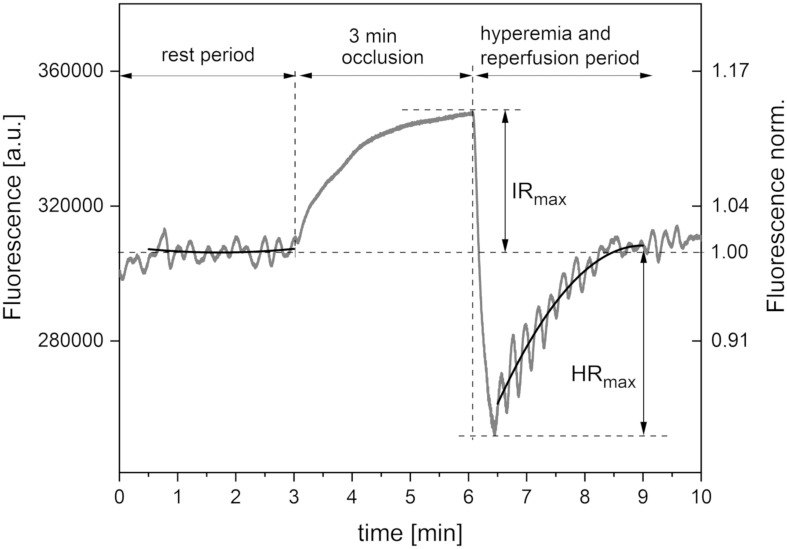
Exemplary FMSF trace recorded for a healthy volunteer (male, age 35 years). Black lines – second order polynomial baselines (see detailed explanation in the text).

Figure 1 presents a typical trace for a healthy individual, recorded using the FMSF method. The baseline was collected for 3 min, following which the occlusion cuff was inflated to 60 mmHg above systolic blood pressure. This caused an increase in NADH fluorescence, known as the ischemic response (IR). After 3 min, the cuff pressure was released and the NADH fluorescence fell below the baseline, reaching a minimum followed by a return to the baseline. This is known as the hyperemic response (HR). In fact, two distinct phases can be identified in HR. The first phase of about 20–30 s is associated with a sharp drop in NADH fluorescence, and can be linked to hyperemia. The post-hyperemic phase is followed by reperfusion, when NADH fluorescence returns to the baseline. The measurement conditions and parameters have been described in detail elsewhere (Piotrowski et al., 2016; Katarzynska et al., 2019b).

The FMSF technique has been used with the post-occlusive reactive hyperemia (PORH) test to evaluate vascular circulation and metabolic regulation in type 1 diabetes (DM1) patients (Katarzynska et al., 2019a). Based on analysis of the FMSF parameters, it was found that DM1 patients with low levels of circulatory disturbance may suffer from serious metabolic deregulation. It is important to note that the FMSF-PORH test can be used to evaluate dysfunctions in both vascular circulation and metabolic regulation. It should be stressed that both macro- and microvessels contribute to oxygen transport to the skin. As the PORH test disrupts blood flow through macro- and microvessels, the response from the macrocirculation can dominate over the response from the microcirculation. This supports our observation that the FMSF-PORH test applied to type 2 diabetes (DM2) patients enables effective monitoring of vascular complications related to cardiovascular disease (CVD). The FMSF-PORH test indicated pronounced dysfunction of circulatory status in DM2 patients, but with relatively preserved metabolic regulation (Katarzynska et al., 2020).

In this paper, we present an analysis of skin flowmotion monitored by the FMSF technique. As there is a very low noise in the recorded FMSF traces, flowmotion can be observed very distinctly and precisely. Two different periods of oscillations can be distinguished in the FMSF signal: basal oscillations at rest, and flowmotion during reperfusion stage. During the occlusion stage, when blood flow in the patient’s limb is blocked, no oscillations are observed. Two quantitative measures of oscillations are defined: FM – a parameter characterizing basal flowmotion at rest; FM(R) – a parameter representing flowmotion during the reperfusion phase. The FM(R) parameter reflects increased microvascular perfusion in response to hypoxia. This process plays a significant role in the physiological adaptation of Sherpa highlanders to hypobaric hypoxia, for example, and of sportsmen to long periods of exertion (Davies et al., 2018; Pickering and Kiely, 2018; Salvi et al., 2018; Carey et al., 2019). Here, disturbed flowmotion in response to hypoxia is identified and characterized in the case of a group of healthy individuals. Special attention is given to the effects of various variables on both the FM and FM(R) parameters. The effects of age, blood pressure, and the oscillation frequency distribution on flowmotion are discussed.

## Materials and Methods

### Assessment of Flowmotion and Measurement Protocol

AngioExpert (Angionica Ltd.) measures changes in the NADH fluorescence signal at 460 nm (excitation at 340 nm) from non-hairy skin on the forearm of a patient. It has been found that measurement of skin NADH fluorescence under resting conditions (without any mechanical, physical, or pharmacological blockage of blood flow) can be successfully used for the assessment of spontaneous oscillations in skin microcirculation (flowmotion). Due to the low frequency of endothelial activity (0.008–0.021 Hz), the shortest time necessary to measure changes in fluorescence is around 2 min, and the optimal length of time is about 5 min. Although there is no time limit for FMSF measurements, the requirement for the patient to remain still during the examination makes 5 min optimal, to avoid artifacts and resulting errors in the estimation of the magnitude of flowmotion. Furthermore, it is advisable to discard the initial section of the measurement (i.e., the first 30–60 s, Figure 1), during which the patient adjusts to the examination conditions. On the other hand, as the reperfusion period following cuff occlusion lasts about 3 min, there is limited time available for the estimation of flowmotion during the reperfusion stage. There is also a delay (of several seconds) between the release of the cuff and the point where maximal hyperemia is reached. The data are then analyzed using dedicated FM analytical software installed on the AngioExpert device, or with commercially available programs such as OriginPro.

The FMSF signal is normalized with respect to the mean value for fluorescence in the central period of measurement (1–2 min). Using this approach, any perturbation due to skin variations, such as skin pigmentation or differences in the fluorescence levels of patients, is minimized, as the relative change from the baseline is normalized (Figure 1). In most cases, the baseline around which the FMSF signal oscillates (corresponding to the average fluorescence characteristic of a given patient at rest) is straight. However, analysis of the FMSF signals collected for a group of patients and healthy volunteers revealed that the baseline at rest is not always horizontal, but can be ascending, descending, ascending to a plateau, descending to a plateau, parabolic, etc. Moreover, since during hyperemia there is a sharp drop in NADH fluorescence followed by a return to the basal level during the reperfusion stage, oscillations are always observed around the ascending line reaching a plateau. It is therefore reasonable to fit the baselines using the polynomial regression method. It has been found that a second order polynomial is flexible enough to fit all fluorescence signals observed at rest and during the reperfusion period which are not related to blood flow oscillations.

In this study, the FMSF signal was collected using the AngioExpert device at a sampling frequency of 25 Hz. An interval of 150 s was selected for analysis of oscillations during the reperfusion period. The second order polynomial was fitted to experimental points in all analyzed cases, using the least squares method (Figure 1, black lines). Although polynomial regression fits a non-linear line to the data, as a statistical problem it remains linear, as the regression function is linear in the unknown parameters that are estimated from the data. Such fitting is unique, as a result of the solution of the system of equations. In the least squares method, the best fit is considered to be that in which the residual sum of squares (SSE) – the sum of the squares of deviations of the experimental points from the polynomial function – is minimal. At the same time, the SSE is a measure of the fitting error (deviation of the points from the polynomial relationship). In the case of analysis of oscillations, the deviation of the fluorescence signal can be used as a measure of the magnitude of oscillations. This parameter is objective and patient-specific. We distinguish between flowmotion at rest (FM) and flowmotion during the reperfusion period [FM(R)].

In statistical analysis, SSE can be normalized with respect to the number of analyzed experimental points (the measurement time). In other words, we calculate the mean squared error (MSE). This provides an unbiased estimate of deviation from the polynomial relationship. It is therefore possible to compare the oscillations at different time intervals. Defining the oscillation parameters in relation to MSE gives an objective, operator-independent characteristic for the tested patient. As MSE values are extremely low, the FM parameters are defined as the MSE values multiplied by a factor of 10^6^, to keep them in the number range of units to hundreds. As mentioned above, the fluorescence changes are normalized so the FM parameters remain unitless values.

**FIGURE 2:**
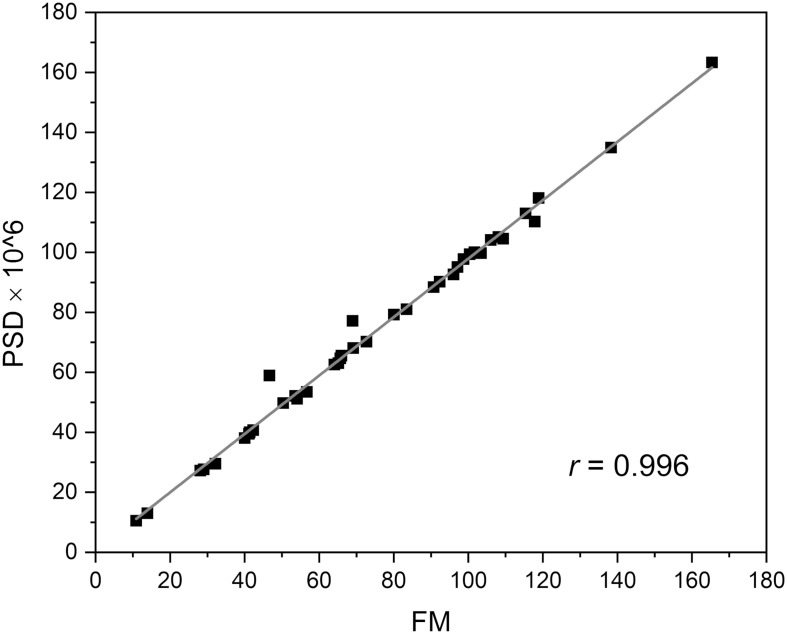
Correlation between power spectra density (PSD × 10^6^) value and FM parameter.

**FIGURE 3:**
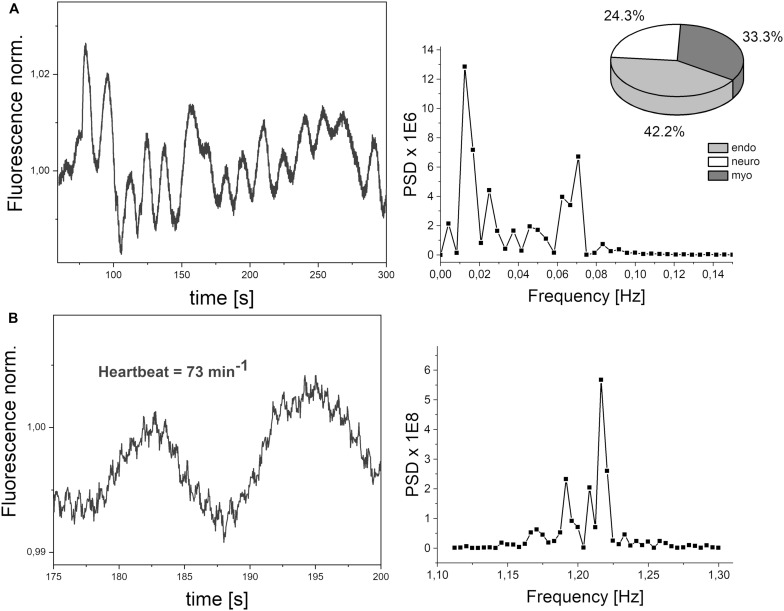
**(A)** Exemplary FMSF signal trace observed for a healthy volunteer (male, age 72 years). Power spectrum density (PSD), calculated a mean squared amplitude of the FFT of the signal. Pie chart shows the presence of three components in flowmotion: endothelial (endo), neurogenic (neuro) and myogenic (myo). **(B)** Heartbeat oscillations seen in the FMSF signal, over a 25 s period of time.

The frequencies of the oscillations contained in the FMSF signal were analyzed using the Fast Fourier Transform (FFT) algorithm. Periodograms were derived from the FFT of the signal, with rectangular windowing and the Power Spectra Density (PSD) calculated as a mean squared amplitude. Although there are windowing functions that more effectively limit the frequency leakage problem associated with the FFT method, we have found that PSD values calculated by rectangular windowing are very well correlated with the FM values, which are also a function of the mean squared amplitude of oscillations (Figure 2). Fast Fourier Transform analysis provides an estimate of the signal power at a given frequency and its relative contribution to the total power of the signal. The calculated power was grouped into three different frequency intervals, namely ≤ 0.021 Hz (0.021–0.052 Hz⟩ and (0.052–0.15 Hz⟩. These are considered to correspond to endothelial, neurogenic and myogenic activities, respectively. The frequency intervals of interest were normalized relative to the total PSD power, to assess the contribution of each frequency to the overall flowmotion, and are presented as pie charts in Figure 3A. Although the hemodynamic effect of heart beat (0.6–2 Hz) can also be seen in the FFT spectra (Figure 3B), it remains weak compared to the total PSD and will not be discussed further. It is, however, worth noticing the clear presence of heartbeat oscillations in the signal, which confirms the high sensitivity of the FMSF method to blood flow oscillations, and the very low noise-to-signal ratio. The limited effect of the heartbeat on changes in total cutaneous NADH fluorescence is very likely the result of the fact that a large fraction of the exciting light (340 nm) does not reach directly the blood vessels, being mainly absorbed by the epidermis (Balu et al., 2013).

### Study Population

The analysis presented in this paper is based on a study described in previous articles (Katarzynska et al., 2019a, 2020) which was conducted at the Medical University of Lodz, Poland, in collaboration with the Medical University of Bialystok and Angionica Ltd. It conformed to the principles outlined in the Declaration of Helsinki and the study protocol was approved by the University Bioethics Committee. All the subjects gave written informed consent prior to participation. The study population consisted of unrelated healthy volunteers from the general population. None of them had a chronic disease or was undergoing treatment. None of the volunteers had clinically abnormal health status.

### Measurement Protocols

Prior to the FMSF measurements, the healthy volunteers underwent an acclimatization at room temperature (for at least 15 min) and their blood pressure was measured. The temperature of the optical window surface in contact with the skin was continuously monitored during the measurement and was usually kept between 27 and 31 degrees. During a single measurement, the change in temperature was not greater than one degree. Two different protocols were used.

#### Protocol 1

The test group consisted of 40 healthy volunteers aged between 31 and 72 (17 males, 23 females). Fluorescence was measured using the FMSF method on the forearm for 5 min, during the resting period only. Each measurement was repeated twice over 1–2 days to estimate the repeatability of the FM parameter and the effects of various oscillations (endo, neuro, myo).

#### Protocol 2

The test group consisted of 35 healthy volunteers aged between 30 and 50 (22 males, 13 females). The participants were enrolled at different clinical centers. Fluorescence was measured using the FMSF method on the left forearm of each patient, over three different periods: a period of at least 3 min for fluorescence baseline measurements; a 3 min occlusion period caused by inflating a cuff placed on the left upper arm to 60 mm Hg above systolic blood pressure; a period of hyperemia/reperfusion lasting at least 3 min following cuff deflation, during which fluorescence returned to the baseline level. Each measurement was repeated within two consecutive days under the same experimental conditions.

### Statistical Analysis

Quantitative data were presented as the mean and the standard deviation. The within-subject coefficient of variation (CV) was calculated as the relative standard deviation. Repeatability of measurements was expressed by the Pearson correlation coefficient (if the Shapiro-Wilk test could not reject normality), or by the Spearman correlation coefficient (if the Shapiro-Wilk test rejected normality). A paired-sample Wilcoxon signed rank test was used to compare the FMSF parameters recorded for different groups of subjects. All reported probability values were two-tailed and a *p* < 0.05 was considered statistically significant.

## Results

### Protocol 1

In this group, only basal flowmotion was analyzed. The demographic and clinical characteristics of the studied population are summarized in Table 1.

**TABLE 1:** Characteristic of the studied population.

Group	Protocol 1	Protocol 2
Male/female (n/n)	17/23	22/13
Age (years)	50.5 ± 12.9	38.4 ± 6.4
Body mass index (BMI) (kg/m^2^)	25.3 ± 4.3	24.8 ± 3.1
Systolic blood pressure (SBP) (mm Hg)	125.8 ± 14.8	124.2 ± 11.9
Diastolic blood pressure (DBP) (mm Hg)	81.5 ± 8.8	79.4 ± 7.7

Figure 3A shows a typical fluorescence signal collected over 5 min. Defined as the mean squared error (multiplied by a factor of 10^6^), FM is the mean fluctuation of the fluorescence from the baseline and thus an estimation of flowmotion oscillations. Low frequency changes are characterized by the presence of three components: endothelial, neurogenic, and myogenic. These are accompanied by weaker blood flow oscillations, caused by a heartbeat with a frequency of around 1 Hz, as shown clearly in the presented signal (see Figure 3B).

The average within-subject coefficient of variation of FM found for the group was 20% and the repeatability of the measurements was good (Pearson’s coefficient *r* = 0.66, *p* = 3 × 10^–6^).

**FIGURE 4:**
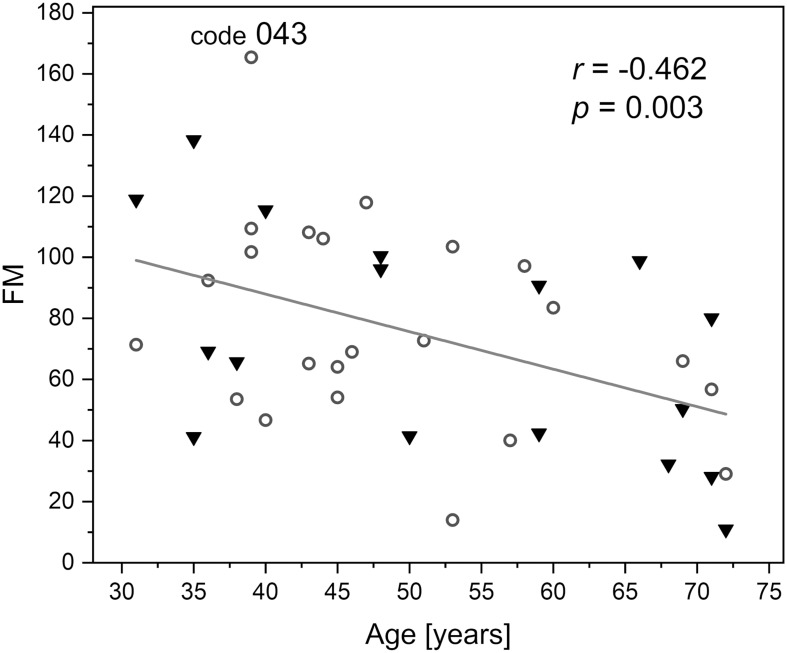
Correlation between the basal flowmotion parameter FM and age (protocol 1, females – circles and males – triangles). Code 043 denotes a result FM = 165 above the upper prediction band (female, age 39 years).

Flowmotion (FM) is age dependent (Pearson’s coefficient *r* = −0.462, *p* = 0.003), but remained similar in our study for males and females. The FM for the whole studied group is shown Figure 4. It is easy to identify individuals with low or strong flowmotion. One result (code 043, female, 39 years old, FM = 165) lies above the upper prediction band (at 95% confidence level). For this woman, both measurements gave relatively high FM values (144 and 187). On the other hand, none of the points can be considered as too low. A negative relationship between skin microcirculatory fluctuations (flowmotion) and age has been reported among a healthy population (Bernardi et al., 1997; Khalil et al., 2016).

### Protocol 2

In this group, the full FMSF measurement procedure was applied. The demographic and clinical characteristic of the studied population are summarized in Table 1. The baseline was collected for 3 min, following which the occlusion cuff was inflated to 60 mmHg above systolic blood pressure, blocking blood flow in the forearm. After 3 min, the cuff was released and the NADH fluorescence fell below the baseline, reaching a minimum followed by a return to the baseline during the reperfusion period. As the hyperemia and reperfusion phase lasts about 3 min, an interval of 150 s was selected for oscillation analysis. For comparison, the same time interval was analyzed on the baseline before occlusion. Two flowmotion parameters were determined for the group, FM and FM(R).

Because of the much shorter time of analysis, the average within-subject coefficient of variation in FM found for the group was 43%. As a consequence, the repeatability of the measurements was diminished, but can still be considered as a fair (Spearman’s coefficient *r* = 0.594, *p* = 2 × 10^–4^). Similar values were obtained for the FM(R) parameter (oscillations during the reperfusion stage): the average coefficient of variation was 41%, Spearman’s coefficient *r* = 0.440, *p* = 0.008.

**FIGURE 5:**
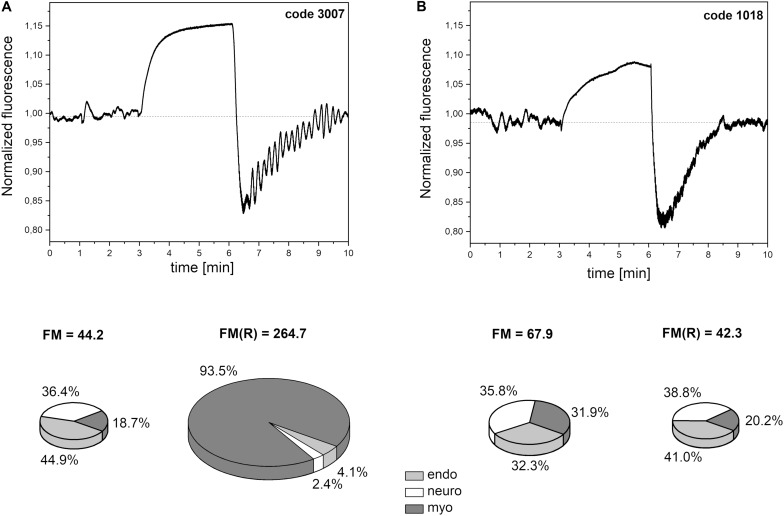
Exemplary FMSF traces recorded for: **(A)** a patient with high flowmotion response to hypoxia (male, age 30 years), **(B)** a patient with low flowmotion response to hypoxia (male, age 34 years). Calculated flowmotion parameters and contribution of different frequency activities to flowmotion are shown below the traces.

In most cases, the changes in skin fluorescence during the reperfusion stage compared to the rest period had a higher frequency and amplitude. A typical fluorescence signal is shown in Figure 5A. The frequency of the observed changes is typical for myogenic oscillations. Activation of such oscillations is often seen in healthy individuals in cases of hypoxia (Salvi et al., 2018; Bugaj et al., 2019). We believe that the activated myogenic oscillations visible in Figure 5A indicate a compensatory effect of unknown origin.

**FIGURE 6:**
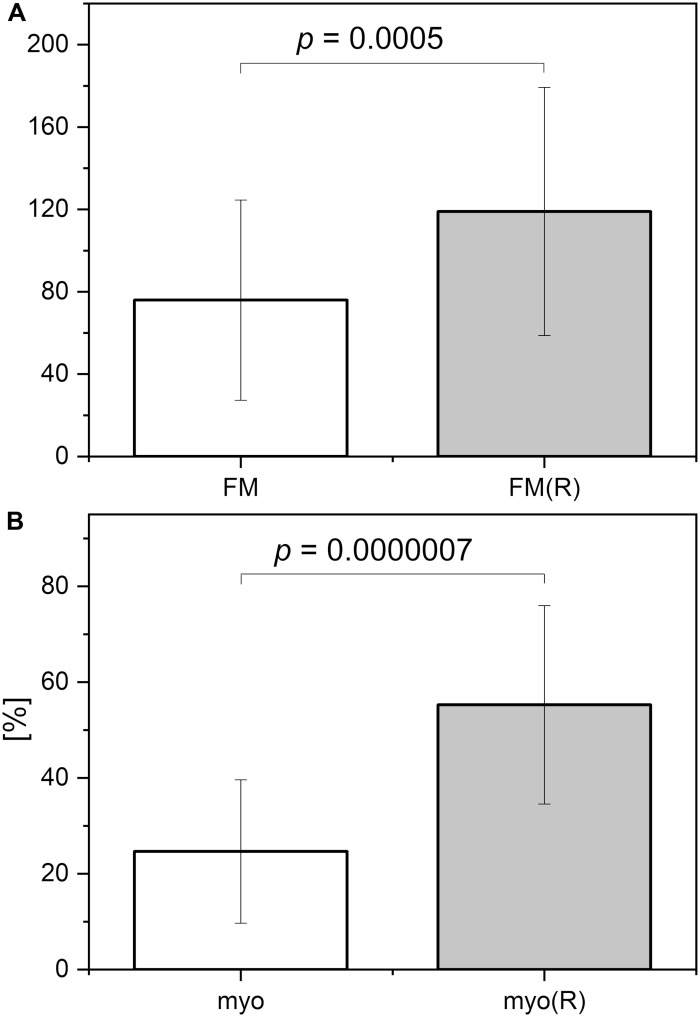
**(A)** Comparison of the flowmotion parameters at rest FM and at reperfusion stage FM(R). **(B)** Contribution of the myogenic component to FM parameter (myo) and FM(R) parameter (myo(R)) (*p*-values were calculated using the paired-sample Wilcoxon signed rank test).

**FIGURE 7:**
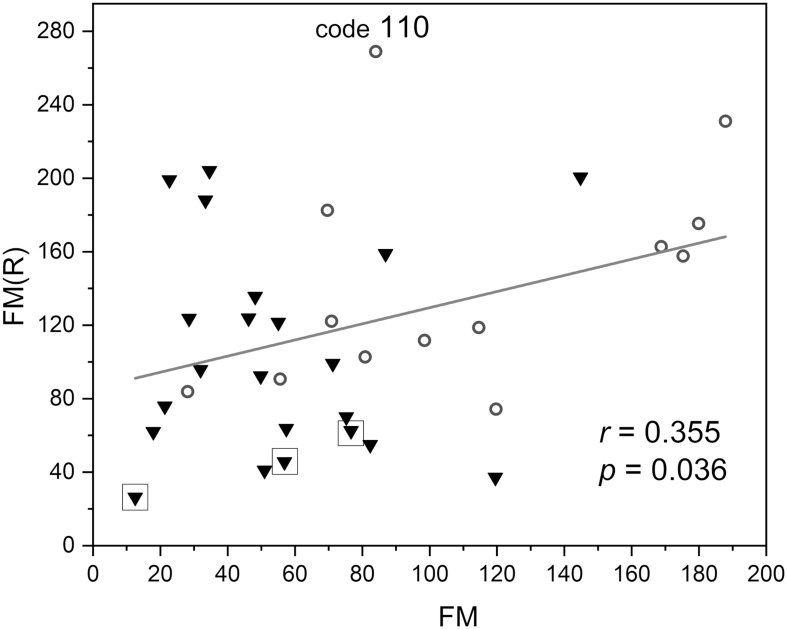
Correlation between the flowmotion parameter at reperfusion stage FM(R) and the basal flowmotion parameter FM (females – circles and males – triangles). Squares denote the individuals with disturbed flowmotion response to hypoxia (shown in Table 2). Code 110 denotes a result FM(R) = 269 above the upper prediction band (female, age 30 years).

**FIGURE 8:**
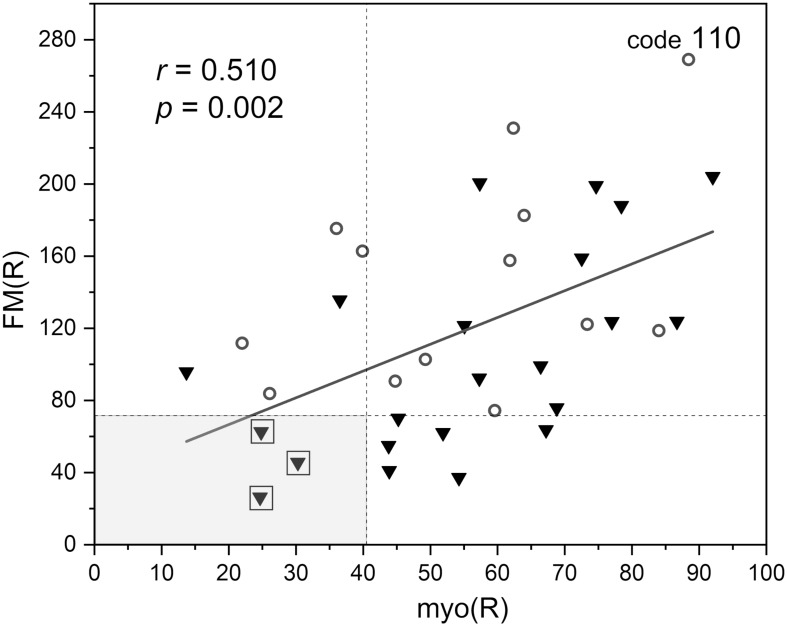
Correlation between the flowmotion parameter at reperfusion stage FM(R) and myogenic component myo(R) (protocol 2, females – circles and males – triangles). Squares denote the individuals with disturbed flowmotion response to hypoxia (shown in Table 2).

In Figure 6, the FM vs. FM(R) flowmotion parameters are compared for the investigated group. The FM(R) parameter is significantly higher, statistically, than the FM parameter. An inverse correlation between FM(R) and age may be observed, but it did not attain statistical significance (*r* = −0.294, *p* = 0.087). The correlation between FM(R) and FM is shown in Figure 7. One result [code 110, female, 30 years old, FM(R) = 269] lies above the upper prediction band (at 95% confidence level). The activation of myogenic oscillations is responsible for the increased flowmotion in response to hypoxia (see Figure 8).

Within the studied group, we selected several individuals for whom both the FM(R) and myo(R) parameters were in the lowest quartile, as shown in Figure 8. These individuals (3 males) seemed to have a disturbed flowmotion response to hypoxia. As shown in Table 2, there was no rise in FM(R) or myo(R) parameters compared to the FM and myo parameters. An exemplary FMSF trace is presented in Figure 5B (code 1018, male, 34 years old). Very low values for FM(R) and myo(R) parameters were observed for diabetic patients, particularly those with ischemic foot ulcers (unpublished results). Such low values seem to indicate impaired adaptation of the microvascular system to hypoxia.

**TABLE 2:** Individuals with disturbed flowmotion response to hypoxia.

Code	Sex	Age (years)	BMI (kg/m^2^)	SBP* (mm Hg)	DBP* (mm Hg)	FM*	FM (R)*	myo* (%)	myo (R)* (%)
2006	M	32	25	127.0	87.5	76.6	62.6	5.6	24.8
1018	M	34	24	118.0	75.0	56.9	45.6	28.7	30.3
1019	M	40	26	127.0	81.5	12.5	26.3	27.8	24.7

* Average values from two measurements.

**FIGURE 9:**
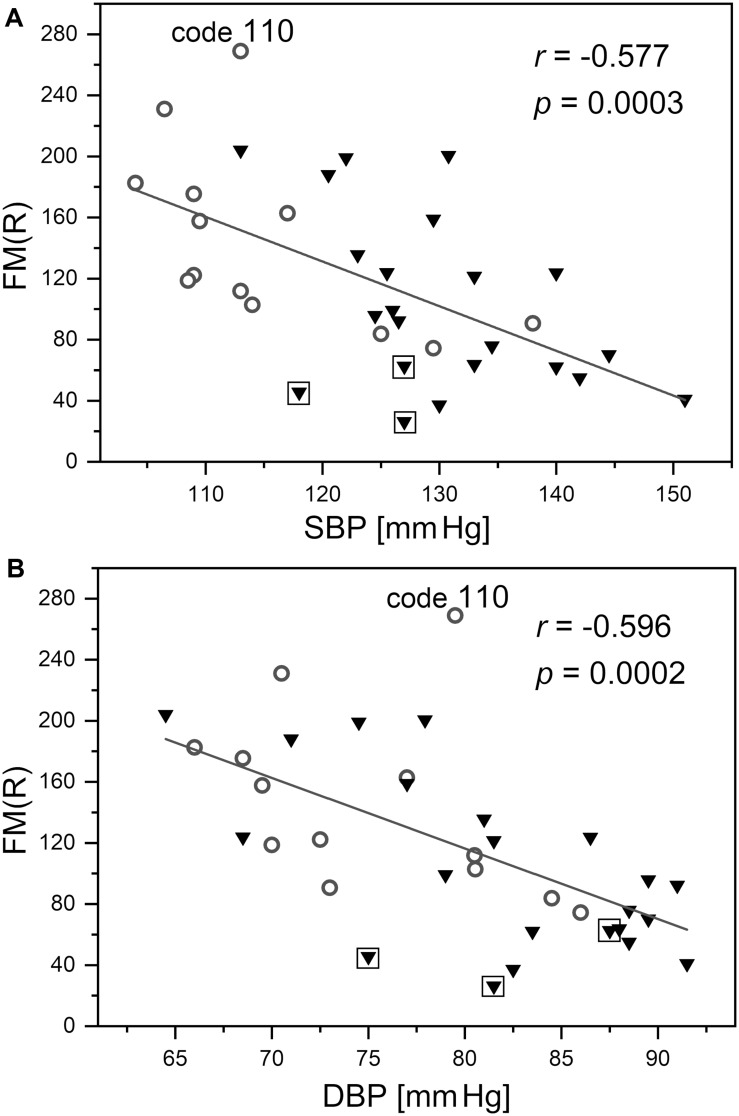
Correlation between the flowmotion parameter at reperfusion stage FM(R) and systolic **(A)** and diastolic **(B)** blood pressure (protocol 2, females – circles and males – triangles). Squares denote the individuals with disturbed flowmotion response to hypoxia (shown in Table 2). Code 110 denotes a result FM(R) = 269 above the upper prediction band (female, age 30 years)

As shown in Figure 9, the FM(R) parameter is strongly inversely correlated with blood pressure. The correlation between the FM parameter and blood pressure did not attain statistical significance (*r* = −0.271, *p* = 0.091).

## Discussion

The FMSF technique is based on monitoring the intensity of NADH emitted from skin tissue on the forearm, the level of which depends on the supply of oxygen delivered by blood circulation. Since, due to physiological factors (predominantly skin pigmentation), direct comparison of absolute levels of fluorescence from the skin is rather useless for the evaluation of circulatory systems, the FMSF method is used in conjunction with the post-occlusive reactive hyperemia (PORH) test.

The FMSF technique appears also to be uniquely suited to the analysis of skin flowmotion and its hypoxia response. Thus, it may be used to predict the microcirculatory status of a patient. The presence of oscillations is a necessary condition for proper functioning of the circulatory system. Although magnitude of flowmotion is very subtle, it can be observed very distinctly and precisely by the FMSF method, due to a very low noise in the recorded FMSF traces and the high sensitivity of the fluorescence-based technique. Two quantitative measures of oscillations can be evaluated from the signal analysis: FM – the flowmotion parameter characterizing basal flowmotion at rest; FM(R) – the flowmotion parameter representing oscillations during the phase of reperfusion.

Monitoring skin flowmotion at rest (before the application of PORH provocation) enables evaluation of skin microcirculatory status. The FM parameter can be used to detect irregularities in flowmotion associated with disease processes, especially cardiovascular diseases (CVD). Therefore, flowmotion analysis based on the FMSF trace at rest could expand the scope of the application of FMSF. Oscillations which are seen directly, even without any additional data processing, may be supported by Fast Fourier Transform analysis, allowing evaluation of the various frequency components that contribute to total flowmotion. It seems likely that patients with low values for flowmotion parameters have abnormal microvascular function. The observation of high myogenic activity during rest periods (in a few cases to levels even higher than those induced by hypoxia) may indicate some dysfunction of the microcirculatory system.

Even more advanced assessment of microcirculation can be obtained by measuring the flowmotion response to hypoxia. The extent of changes in the flowmotion caused by, for example, thermal or pharmacological effects, limited oxygen delivery, or restricted blood flow, can be used as a measure of the ability of the circulatory system to compensate for such effects. Vasomotion has been suggested as an important factor in reducing microvascular resistance and ensuring adequate blood flow in the presence of lower oxygen delivery (Paparde et al., 2015).

The observed correlation between the FM(R) parameter and blood pressure presented in Figure 9 requires special attention. It can be seen that the changes in the FM(R) parameter are very sensitive to both systolic and diastolic blood pressure, measured for healthy individuals with normalized blood pressure. This observation suggests that the flowmotion response to hypoxia was particularly strong in the segment of the studied group with the lowest blood pressure. This identifies blood pressure as a very sensitive physiological parameter, responsible for the response to hypoxia. It has been suggested previously that post-ischemic blood flow oscillations are a sensitive measure of microcirculatory dysfunction in humans with essential arterial hypertension (Rossi et al., 2006a). The FM(R) parameter measured using the FMSF technique combined with PORH provocation allows for early detection of microcirculatory complications associated with elevated blood pressure. Such an approach may have important clinical implications.

A careful analysis of the FM(R) and myo(R) parameters identified three individuals (males) with a disturbed flowmotion response to hypoxia (Table 2). Perhaps not accidently, only males were identified in the quartile with the lowest FM(R) values. This may suggest some differences between females and males in terms of microcirculatory status. The existence of gender differences in vascular function is well known (Thompson and Khalil, 2003; Chen, 2018; Pabbidi et al., 2018) and it is thought that sex hormones play a crucial role. The incidence of cardiovascular disease (CVD) is lower in pre-menopausal women, but increases with age and the onset of the menopause compared to men of a similar age. Due to the small size of the studied group, the analysis presented here does not allow for a rigorous evaluation of the effect of sex on microcirculatory status, which requires further research.

Analysis of flowmotion during the reperfusion stage [FM(R)] makes it possible to identify individuals with disturbed activation of myogenic oscillations. Such analysis may help physically active healthy people to plan their exercise schedule, or with selecting athletes with strong responses to hypoxia, who may be especially suited to endurance sports (long-distance running, climbing, etc.). The flowmotion response to hypoxia evaluated by the FM(R) parameter may also assist in the prognosis of recovery in cases of diseases accompanied by hypoxia.

## Data Availability Statement

The datasets generated for this study are available on request to the corresponding author.

## Ethics Statement

Written, informed consent was obtained from the participating individuals for the publication of any potentially identifiable images or data included in this manuscript.

## Author Contributions

JK performed and analyzed the data and contributed to the manuscript preparation. TC prepared the computational program. LS contributed to the data analysis. AM performed the data and statistical analysis, interpreted the results, and prepared the manuscript. JG designed the study, performed the literature search, and revised the manuscript. All authors contributed to the article and approved the submitted version.

## Conflict of Interest

JK, TC, and LS were employed by Angionica Ltd. JG and AM were inventors of the patents protecting the use of FMSF technology.

The authors declare that the research was conducted in the absence of any commercial or financial relationships that could be construed as a potential conflict of interest.

## References

[B1] Aalkjær C. Boedtkjer D. Matchkov V. (2011). Vasomotion – what is currently thought? *Acta Physiol.* 202 253–269. 10.1111/j.1748-1716.2011.02320.x 21518271

[B2] Balu M. Mazhar A. Hayakawa C. K. Mittal R. Krasieva T. B. König K. (2013). In vivo multiphoton NADH fluorescence reveals depth-dependent keratinocyte metabolism in human skin. *Biophys. J.* 104 258–267. 10.1016/j.bpj.2012.11.3809 23332078 PMC3540245

[B3] Bari F. Tóth-Szûki V. Domoki F. Kálmán J. (2005). Flow motion pattern differences in the forehead and forearm skin: age-dependent alterations are not specific for Alzheimer’s disease. *Microvasc. Res.* 70 121–128. 10.1016/j.mvr.2005.09.001 16229865

[B4] Bernardi L. Rossi M. Leuzzi S. Mevio E. Fornasari G. Calciati A. (1997). Reduction of 0.1 Hz microcirculatory fluctuations as evidence of sympathetic dysfunction in insulin-dependent diabetes. *Cardiovasc. Res.* 34 185–191. 10.1016/S0008-6363(97)00017-5 9217889

[B5] Bernjak A. Clarkson P. B. M. McClintock P. V. E. Stefanovska A. (2008). Low-frequency blood flow oscillations in congestive heart failure and after beta1-blockade treatment. *Microvasc. Res.* 76 224–232. 10.1016/j.mvr.2008.07.006 18721820 PMC2666799

[B6] Bruning R. S. Kenney W. L. Alexander L. M. (2015). Altered skin flowmotion in hypertensive humans. *Microvasc. Res.* 97 81–87. 10.1016/j.mvr.2014.01.001 24418051 PMC5466804

[B7] Bugaj O. Zieliński J. Kusy K. Kantanista A. Wieliński D. Guzik P. (2019). The effect of exercise on the skin content of the reduced form of NAD and its response to transient ischemia and reperfusion in highly trained athletes. *Front. Physiol.* 10:600. 10.3389/fphys.2019.00600 31156467 PMC6529559

[B8] Carey D. Thanaj M. Davies T. Gilbert-Kawai E. Mitchell K. Levett D. Z. H. (2019). Enhanced flow-motion complexity of skin microvascular perfusion in Sherpas and lowlanders during ascent to high altitude. *Sci. Rep.* 9:14391. 10.1038/s41598-019-50774-0 31591502 PMC6779732

[B9] Chen H. (2018). Mechanism of gender-related differences in vascular function. *Cardiovasc. Pharmacol. Open Access* 7:4 10.4172/2329-6607.1000246

[B10] Clough G. F. Kuliga K. Z. Chipperfield A. J. (2017). Flow motion dynamics of microvascular blood flow and oxygenation: evidence of adaptive changes in obesity and type 2 diabetes mellitus/insulin resistance. *Microcirculation* 24 1–13. 10.1111/micc.12331 27809397

[B11] Cole W. C. Gordon G. R. Braun A. P. (2019). “‘Cellular and Ionic Mechanisms of Arterial Vasomotion’,” in *Smooth Muscle Spontaneous Activity: Physiological and Pathological Modulation*, eds Hashitani H. Lang R. J. (Singapore: Springer Singapore), 297–312. 10.1007/978-981-13-5895-1_12 31183832

[B12] Davies T. Gilbert-Kawai E. Wythe S. Meale P. Mythen M. Levett D. (2018). Sustained vasomotor control of skin microcirculation in Sherpas versus altitude-naive lowlanders: experimental evidence from Xtreme Everest 2. *Exp. Physiol.* 103 1494–1504. 10.1113/EP087236 30182473

[B13] Katarzynska J. Borkowska A. Czajkowski P. Los A. Szczerbinski L. Milewska-Kranc A. (2019a). Flow mediated skin fluorescence technique reveals remarkable effect of age on microcirculation and metabolic regulation in type 1 diabetes. *Microvasc. Res.* 124 19–24. 10.1016/j.mvr.2019.02.005 30807771

[B14] Katarzynska J. Borkowska A. Los A. Marcinek A. Cypryk K. Gebicki J. (2020). Flow-mediated skin fluorescence (FMSF) technique for studying vascular complications in type 2 diabetes. *J. Diabetes Sci. Technol.* 14 693–694. 10.1177/1932296819895544 31855069 PMC7576949

[B15] Katarzynska J. Lipinski Z. Cholewinski T. Piotrowski L. Dworzynski W. Urbaniak M. (2019b). Non-invasive evaluation of microcirculation and metabolic regulation using flow mediated skin fluorescence (FMSF): technical aspects and methodology. *Rev. Sci. Instrum.* 90:104104 10.1063/1.5092218

[B16] Khalil A. Humeau-Heurtier A. Gascoin L. Abraham P. Mahé G. (2016). Aging effect on microcirculation: a multiscale entropy approach on laser speckle contrast images. *Med. Phys.* 43 4008–4016. 10.1118/1.4953189 27370119

[B17] Kvandal P. Landsverk S. A. Bernjak A. Stefanovska A. Kvernmo H. D. Kirkebøen K. A. (2006). Low-frequency oscillations of the laser doppler perfusion signal in human skin. *Microvasc. Res.* 72 120–127. 10.1016/j.mvr.2006.05.006 16854436

[B18] Mizeva I. Makovik I. Dunaev A. Meglinski I. (2017). Analysis of skin blood microflow oscillations in patients with rheumatic diseases. *J. Biomed. Opt.* 22 1–3. 10.1117/1.JBO.22.7.070501 28703257

[B19] Nilsson H. Aalkjaer C. (2003). Vasomotion: mechanisms and physiological importance. *Mol. Interv.* 3 79–78. 10.1124/mi.3.2.79 14993429

[B20] Pabbidi M. R. Kuppusamy M. Didion S. P. Sanapureddy P. Reed J. T. Sontakke S. P. (2018). Sex differences in the vascular function and related mechanisms: role of 17β-estradiol. *Am. J. Physiol. Circ. Physiol.* 315 H1499–H1518. 10.1152/ajpheart.00194.2018 30192631

[B21] Paparde A. Plakane L. Circenis K. Aivars J. I. (2015). Effect of acute systemic hypoxia on human cutaneous microcirculation and endothelial, sympathetic and myogenic activity. *Microvasc. Res.* 102 1–5. 10.1016/j.mvr.2015.07.005 26211848

[B22] Pedanekar T. Kedare R. Sengupta A. (2019). Monitoring tumor progression by mapping skin microcirculation with laser doppler flowmetry. *Lasers Med. Sci.* 34 61–77. 10.1007/s10103-018-2600-z 30141135

[B23] Pickering C. Kiely J. (2018). Exercise response efficiency: a novel way to enhance population health? *Lifestyle Genomics* 11 129–135. 10.1159/000501206 31302657

[B24] Piotrowski L. Urbaniak M. Jedrzejczak B. Marcinek A. Gebicki J. (2016). Note: flow mediated skin fluorescence - A novel technique for evaluation of cutaneous microcirculation. *Rev. Sci. Instrum.* 87 3–6. 10.1063/1.4945044 27036844

[B25] Rossi M. Carpi A. Di Maria C. Galetta F. Santoro G. (2006a). Spectral analysis of laser Doppler skin blood flow oscillations in human essential arterial hypertension. *Microvasc. Res.* 72 34–41. 10.1016/j.mvr.2006.04.001 16797604

[B26] Rossi M. Carpi A. Galetta F. Franzoni F. Santoro G. (2006b). The investigation of skin blood flowmotion: a new approach to study the microcirculatory impairment in vascular diseases? *Biomed. Pharmacother.* 60 437–442. 10.1016/j.biopha.2006.07.012 16935461

[B27] Rossi M. Matteucci E. Pesce M. Consani C. Galetta F. Giampietro O. (2013). Study of skin vasomotion in type 1 diabetic patients and of its possible relationship with clinical and laboratory variables. *Clin. Hemorheol. Microcirc.* 53 357–367. 10.3233/CH-2012-1621 23070199

[B28] Salvi P. Faini A. Castiglioni P. Brunacci F. Montaguti L. Severi F. (2018). Increase in slow-wave vasomotion by hypoxia and ischemia in lowlanders and highlanders. *J. Appl. Physiol.* 125 780–789. 10.1152/japplphysiol.00977.2017 29927733

[B29] Schmidt-Lucke C. Borgström P. Schmidt-Lucke J. A. (2002). Low frequency flowmotion/(vasomotion) during patho-physiological conditions. *Life Sci.* 71 2713–2728. 10.1016/S0024-3205(02)02110-0 12383879

[B30] Sorelli M. Francia P. Bocchi L. De Bellis A. Anichini R. (2019). Assessment of cutaneous microcirculation by laser doppler flowmetry in type 1 diabetes. *Microvasc. Res.* 124 91–96. 10.1016/j.mvr.2019.04.002 30959000

[B31] Stefanovska A. Bracic M. Kvernmo H. D. (1999). Wavelet analysis of oscillations in the peripheral blood circulation measured by laser doppler technique. *IEEE Trans. Biomed. Eng.* 46 1230–1239. 10.1109/10.790500 10513128

[B32] Thompson J. Khalil R. A. (2003). Gender differences in the regulation of vascular tone. *Clin. Exp. Pharmacol. Physiol.* 30 1–15. 10.1046/j.1440-1681.2003.03790.x 12542447

[B33] Ticcinelli V. Stankovski T. McClintock P. V. E. Stefanovska A. (2015). “Ageing of the couplings between cardiac, respiratory and myogenic activity in humans,” in *2015 37th Annual International Conference of the IEEE Engineering in Medicine and Biology Society (EMBC)*, Milan, 7366–7369. 10.1109/EMBC.2015.7320093 26737993

[B34] Tikhonova I. V. Kosyakova N. I. Tankanag A. V. Chemeris N. K. (2016). Oscillations of skin microvascular blood flow in patients with asthma. *Microcirculation* 23 33–43. 10.1111/micc.12252 26494289

